# Cerebral Correlates of Abnormal Emotion Conflict Processing in Euthymic Bipolar Patients: A Functional MRI Study

**DOI:** 10.1371/journal.pone.0134961

**Published:** 2015-08-05

**Authors:** Pauline Favre, Mircea Polosan, Cédric Pichat, Thierry Bougerol, Monica Baciu

**Affiliations:** 1 Univ. Grenoble Alpes, LPNC, CNRS UMR 5105, Grenoble, France; 2 CHU de Grenoble, Pôle Psychiatrie et Neurologie, Centre Expert en Troubles Bipolaires, Université Joseph Fourier, Grenoble, France; 3 Univ. Grenoble Alpes, GIN, INSERM, CHU de Grenoble, Grenoble, France; University of Frankfurt, GERMANY

## Abstract

**Background:**

Patients with bipolar disorder experience cognitive and emotional impairment that may persist even during the euthymic state of the disease. These persistent symptoms in bipolar patients (BP) may be characterized by disturbances of emotion regulation and related fronto-limbic brain circuitry. The present study aims to investigate the modulation of fronto-limbic activity and connectivity in BP by the processing of emotional conflict.

**Methods:**

Fourteen euthymic BP and 13 matched healthy subjects (HS) underwent functional magnetic resonance imaging (fMRI) while performing a word-face emotional Stroop task designed to dissociate the monitoring/generation of emotional conflict from its resolution. Functional connectivity was determined by means of psychophysiological interaction (PPI) approach.

**Results:**

Relative to HS, BP were slower to process incongruent stimuli, reflecting higher amount of behavioral interference during emotional Stroop. Furthermore, BP showed decreased activation of the right dorsolateral prefrontal cortex (DLPFC) during the monitoring and a lack of bilateral amygdala deactivation during the resolution of the emotional conflict. In addition, during conflict monitoring, BP showed abnormal positive connectivity between the right DLPFC and several regions of the default mode network.

**Conclusions:**

Overall, our results highlighted dysfunctional processing of the emotion conflict in euthymic BP that may be subtended by abnormal activity and connectivity of the DLPFC during the conflict monitoring, which, in turn, leads to failure of amygdala deactivation during the resolution of the conflict. Emotional dysregulation in BP may be underpinned by a lack of top-down cognitive control and a difficulty to focus on the task due to persistent self-oriented attention.

## Introduction

Bipolar disorder (BD) is a severe mood disorder characterized by alternating manic and depressed periods that could be punctuated by inter-critic intervals, without extreme mood symptoms, namely, the euthymic periods. Residual symptoms of euthymic states are especially characterized by enhanced emotional reactivity [1, 2] and executive functions deficits [3, 4] that would reflect trait abnormalities of the illness. Indeed, numerous neuropsychological studies highlighted persistent impairments in emotional and cognitive processes in euthymic BP but the cerebral correlates and the mechanisms of these impairments are still unclear [5, 6].

In the past decades, functional magnetic resonance imaging (fMRI) studies that have sought to identify brain abnormalities in BP pointed out impairment of frontal and limbic regions during both emotional and cognitive tasks [7–9], and suggest emotional regulation and homeostasis disturbances [5, 6, 10]. Phillips et al.’s model of automatic and voluntary emotional regulation [10], highlighted abnormalities within the ventral system in BP, specifically the left ventromedial prefrontal cortex (VMPFC), which may be responsible for automatic emotion dysregulation in BP. To investigate emotional regulation sub-processes, different cognitive paradigms have been proposed. Among them, emotional Stroop tasks were used to examine neural systems implicated in the automatic attentional control of emotion. In the traditional emotional Stroop task participants are required to identify the color of written words (or the number of stimuli in the counting Stroop version) that could be either neutral or emotionally salient. Thus, the traditional emotional Stroop assesses the ability of emotional information to implicitly divert the attention from the main task, not the interference *per se*. In healthy subjects (HS), that task has been associated, inter alia, with activation of left rostral anterior cingulate cortex (ACC) [11], while the counting Stroop version recruited a larger network including rostral ACC, as well as, dorsolateral (DLPFC), dorsomedial (DMPFC) and orbitofrontal (OFC) prefrontal cortices [12]. Compared to HS, euthymic BP showed decreased VMPFC activity during emotional Stroop tasks [13, 14].

A modified emotional Stroop paradigm allowing the direct assessment of the emotional conflict processing, similarly to the original color-word Stroop task [15], was recently developed and known as ‘word-face emotional Stroop’ [16]. This task consists of identifying the emotional expression of fearful and joyful faces while ignoring the emotional word, which can be either congruent or incongruent with the facial expression (i.e., “Fear” or “Happy”). Using this task, we previously showed decreased activity of the ventrolateral prefrontal cortex (VLPFC) in euthymic BP in comparison to HS [17]. By using a similar task, another recent study also revealed decreased lateral prefrontal activity while processing emotional conflict in hypomanic, depressive and euthymic BP. [18] In addition, the later study, showed that in response to incongruent trials and unlike the HS, euthymic BP exhibited *deactivation* of several areas belonging to the default mode network (DMN) including the rostral ACC during euthymiathe hippocampus during depression and posterior cingulate cortex (PCC) during hypomania, which was attributed to exaggerated disengagement of internal thoughts and memories.

Otherwise, it has been suggested that the sequence of congruent and incongruent trials should also be considered during Stroop tasks. Indeed, it was demonstrated that the processing of incongruent trials is faster when preceded by incongruent trials but slower if preceded by congruent trials [19, 20]. Consequently, the conflict generated in response to incongruent trial might activate a proactive cognitive control mechanism [21], which facilitates the resolution of the conflict in the next incongruent trial [22]. Then, incongruent stimuli could further be subdivided into two types, high conflict resolution trials (i.e., incongruent stimuli preceded by incongruent stimuli—HR), and low conflict resolution trials (i.e., incongruent stimuli preceded by congruent stimuli—LR). The distinction between these two types of trials allows to assess separately the *conflict monitoring* (or generation), which may lead to increased cerebral activation in response to LR in comparison to HR trials from the *resolution* of the conflict reflected by the increased activation for HR in comparison to LR trials [22]. In HS, Etkin et al. [16] revealed greater for HS involvement of the amygdala, the DLPFC and DMPFC during emotional conflict monitoring, while conflict resolution was rather associated with activation of the rostral ACC. In addition, the authors showed that activation of the rostral ACC was predicted by prefrontal activation during monitoring and was accompanied by simultaneously decreased activity of the amygdala. This suggests that emotional conflict is resolved by a top-down cognitive control exerted by prefrontal areas on the amygdala.

In the current study, we aimed at investigating automatic emotion regulation processing in euthymic BP by using an emotional Stroop paradigm conceived to distinguish the monitoring from the resolution of emotional conflict. We assume that disturbances of emotion regulation processing reported previously in BD could result from a lack of prefrontal-cognitive top-down control on limbic-emotional areas. We hypothesized that compared to HS, euthymic BP would show (a) decreased activation of the lateral prefrontal cortex during the monitoring of the emotional conflict and (b) decreased activation of the rostral ACC and increased activation of the amygdala during the resolution of the emotional conflict. Based on previous results [18, 23] we also expect revealing (c) abnormal activity and connectivity between prefrontal and limbic regions as well as with those who belong to the DMN.

## Materials and Methods

### Participants

Fourteen euthymic BP (mean of age ± SD: 44.1 ± 9.6 years, 8 females) and 13 HS matched on age and gender (mean of age 44.1 ± 10.8 years, 9 females) were included in the study. All participants were right-handed and had equivalent education level. Mood symptoms in BP were evaluated with the Montgomery and Asberg Depression Rating Scale (MADRS) [24, 25] and Young Mania Rating Scale (YMRS) [26, 27]. Patients were included in the study if they reported having been euthymic for at least one month prior to scanning and if they had an MADRS’s score < 15 and an YMRS’s score < 7 (Table 1). All patients were diagnosed for bipolar disorder (BD) by an experienced psychiatrist according to DSM IV criteria for BD and confirmed by using the French version of the Structured Clinical Interview (SCID) for DSM IV [28]. Five BD patients were diagnosed with type I BD, six with type II and two with “not otherwise specified” BD type. The mean age of illness onset was 28.0 ± 9.0 years and the mean duration of the illness was 16.1 ± 11.1 years. Two patients were medication-free and the others received different combinations of drugs including lithium (64%), anticonvulsants (43%), antidepressants (21%) and atypical antipsychotic agents (7%).

**Table 1:** Demographic and clinical information for healthy subjects and euthymic bipolar patients.

	BP		HS	
	Mean	SD	Mean	SD
Age (years)	44.07	9.63	44.08	10.85
Gender (% female)	64%	-	69%	-
Age of illness onset (years)	27.96	8.96	-	-
Duration of the illness (years)	16.08	11.10	-	-
Montgomery and Asberg Depression Rating Scale (MADRS)	7.61	5.01	-	-
Young Mania Rating Scale (YMRS)	2.92	3.17	-	-
Past depressive episodes	4.00	3.55	-	-
Past hypomanic episodes	4.00	3.21	-	-
Past manic episodes	1.75	1.49	-	-
Positive history of psychotic symptoms	46%	-	-	-

BP: Bipolar patients; HS: Healthy subjects

For all participants as exclusion criteria we considered: history of alcohol or drug abuse; current or past neurological and/or medical diseases affecting cognition; history of head trauma with loss of consciousness; metal implants. Additionally, we excluded (a) for EBP, any current other Axis I psychiatric disorder and sismotherapy during the precedent year; (b) for HS, past or present psychiatric disorder and family history of psychiatric disorders, as well as any medical treatment affecting cerebral activity. HS were selected and included in the study after interview with a psychiatrist (PI), and according to the SCID. Participants received complete description of the study and they all provided written informed consents.

### Ethic statement

The study was approved by the Grenoble Hospital Ethic Committee (n° AU 898/2011) and is registered on (NCT01821469). Participants received complete description of the study and they all provided written informed consents. All participants were in capacity to give written consent before the beginning of the experiment (as determinate by the PI).

### Stimuli and Task

**Fig 1:**
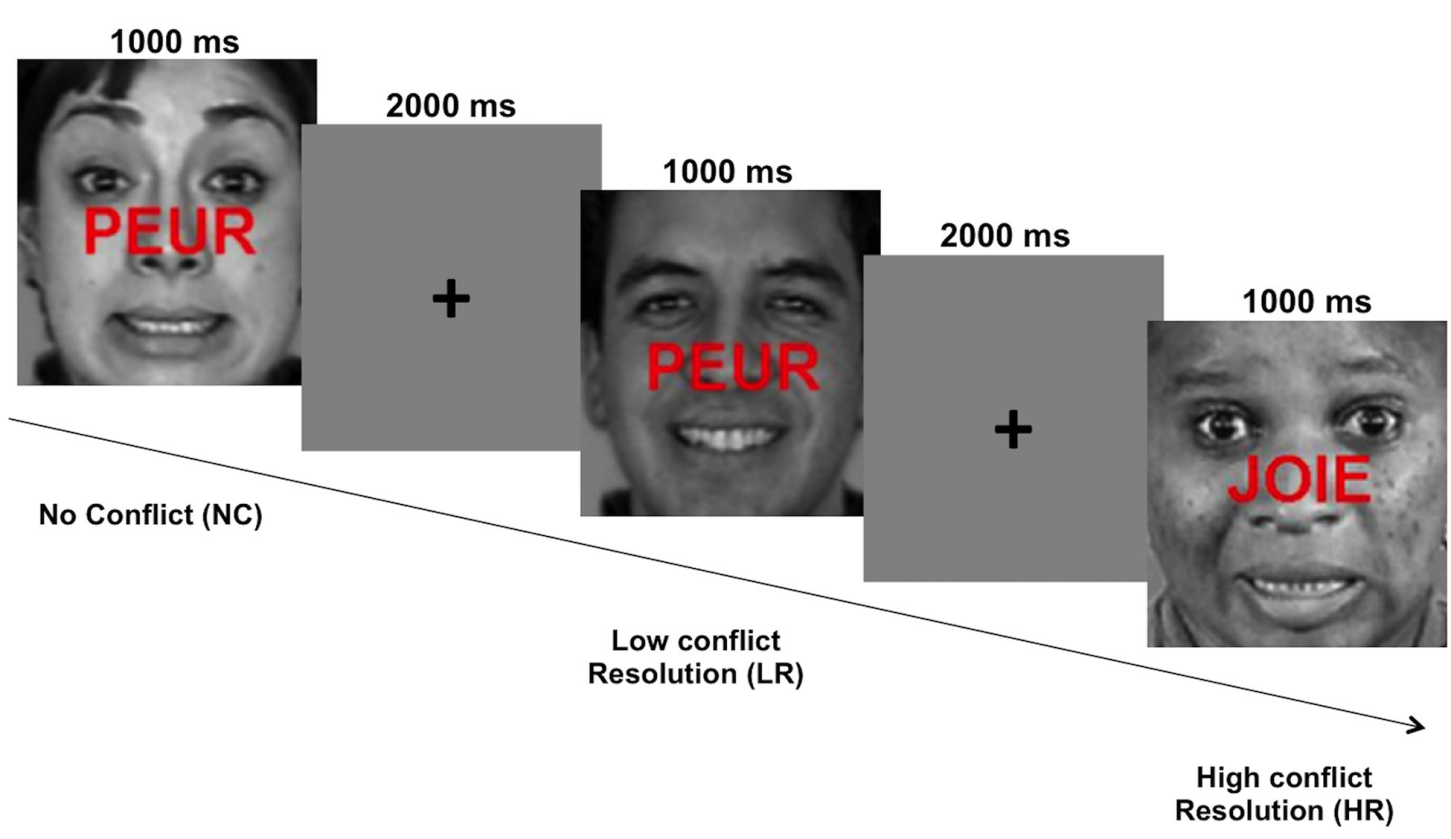
Example of two successive trials presented in word-face emotional Stroop. Stimuli were either congruent or incongruent according to the valence of facial expression (i.e., joyful or fearful) and the valence of the word written across them (i.e., *joie*: happy or *peur*: fear). High conflict resolution trials consisted of incongruent stimuli preceded by incongruent stimuli; Low conflict resolution trials consisted of incongruent stimuli preceded by congruent stimuli; No conflict trials consisted of congruent stimuli preceded by either congruent (NC1) or by incongruent stimuli (NC2) (not shown in the figure). Pictures were extracted from the “Montreal Set of Facial Display of Emotion” (MSFDE) database [30].

We used a modified version of the word-face emotional Stroop task developed by Etkin et al. [16]. In the original version, Etkin et al. [16] used emotional facial stimuli from the “pictures of facial affect” database [29]. Our stimuli were different from those used by Etkin et al. as they were extracted from a more recent database, the “Montreal set of facial display of emotion” (MSFDE) [30]. In order to build the set of stimuli, twenty-five grayscale faces with different identities, expressing happy or fear emotions, were selected. It comprised 12 male and 13 female faces, with African, Asian, Caucasian or Hispanic ethnicity. The French words “*joie*” (happy) or “*peur*” (fear) written in capital letters and in red color were superimposed on the faces to create congruent and incongruent stimuli. Similarly to Etkin et al. [16], we also manipulated the order of the stimuli to create high conflict resolution (HR) and low conflict resolution (LR) trials (Fig 1). According to congruency “current vs. previous trial” we obtained four types of experimental conditions: current incongruent—previous congruent (i.e., LR); current incongruent—previous incongruent (i.e., HR); current congruent—previous congruent (no conflict 1 –NC1); current congruent—previous incongruent (no conflict 2 –NC2). Twenty-five different stimuli per condition were presented (i.e., 100 stimuli for the whole experiment). Features such as identity, gender and origin of the presented faces were randomized within conditions.

To perform the task, participants were instructed to identify as accurately and rapidly as possible the emotional expression of faces while ignoring the associated word. They gave responses by pressing a response key with two buttons with their dominant hand, corresponding to the two possibilities (happy and fear). For each stimuli and each participant, the accuracy (% correct responses) and the response time (ms) were recorded throughout the duration of stimulus presentation and inter-stimulus interval (for 2500 ms duration). Before fMRI acquisition, participants were trained to perform the task with different stimuli that those used during the experiment.

A pseudo-randomized event-related fMRI paradigm was optimized [31] for 100 events and 45 additional null-events. The null-events were added in order to provide appropriate baseline measure [31] and corresponded to a black fixation cross displayed in the center of the grey screen. All conditions were evaluated during one functional run in an fMRI session. The duration of each trial was 3000 ms (1000 ms stimulus presentation and 2000 ms additional fixation cross). The presentation order of stimuli was counterbalanced across participants, according to three possibilities provided by the optimization. The total duration of the run was 7min 25s.

### MRI acquisition

MR images were acquired with a whole-body 3T MR scanner (Achieva 3.0 TX Philips, Grenoble MRI facility IRMaGE). Functional images were acquired with the manufacturer-provided gradient-echo/T2* weighted EPI sequence. Thirty-seven adjacent axial slices parallel to the bi-commissural plane were acquired in interleaved mode. Slice thickness was 3.75 mm. During the functional run, the cerebral volume was measured 174 times. The in-plane voxel size was 3 x 3 mm (216 × 216 mm field of view acquired with 72 × 72 pixels data matrix; reconstructed with to zero filing to 128 × 128 pixels). The main sequence parameters were TR = 2.5s, TE = 30ms and flip angle = 77°. Finally, a T1-weighted high-resolution three-dimensional anatomical volume was acquired by using a turbo field echo (TFE) sequence (field of view = 224 × 256 × 176 mm; resolution: 0.8 × 0.8 × 0.8 mm; acquisition matrix: 280 × 320 × 220 pixels).

### Data analyses

#### Behavioral data analysis

Two separate analyses of variance (ANOVA) were performed, one on accuracy (% correct responses—CR) and another one on the mean response time (RT) for correct responses. The two ANOVAs included the following factors: Condition (NC1, NC2, LR, HR) as a within-subject factor, and Group (BP, HS) as a between-subject factor. Behavioral results are reported at *P* < 0.05 threshold.

#### fMRI data analysis. Preprocessing

fMRI analyses were performed by using the SPM8 software package (Welcome Department of Imaging Neuroscience, Institute of Neurology, London, UK), running on Matlab 7.9 (R2009b) (Mathworks, Natick, MA, USA). Brain regions involved in different contrasts were labeled by means of macroscopic parcellation of the MNI single subject reference brain [32]. Functional images were first time-corrected (slice timing), and realigned using rigid body transformations. The T1-weighted anatomical volume was coregistered to mean image created by the realignment procedure and was normalized to the MNI space using a tri-linear interpolation. The anatomical normalization parameters were then used for the normalization of functional volumes. Finally, all functional images were smoothed using 8-mm full-Width at half maximum Gaussian.

#### First-level analysis

For each participant, experimental conditions (LR, HR, NC1, NC2) were modeled using the General Linear Model (GLM) [33]. The six realignment parameters were also included as covariate of no interest. The blood-oxygen-level dependence response for each event was modeled using a canonical form of the hemodynamic response function (HRF). Before estimation, a high-pass filtering with a cutoff period of 128s was applied. The first level analysis (individual level) consisted of two contrast effects: (1) the conflict *Monitoring* contrast [LR > HR], to identify cerebral regions involved in the monitoring/generation of the emotional conflict; (2) the conflict *Resolution* contrast [HR > LR], to identify cerebral regions involved in the resolution of the emotional conflict.

#### Second-level analysis

At the second level, random-effect between-group analyses were conducted using two-sample *t*-tests [34]. Euthymic BP and HS cerebral activity was compared according to the two main contrasts defined at individual level (i.e., conflict *Monitoring* and *Resolution*). The resulting activation maps were thresholded at *P* < 0.001 (uncorrected) with a minimum cluster size of 10 voxels. Given our *a priori* hypothesis that particular regions would be affected in BP, the groups were also compared using small volume correction (SVC) to the GLM targeting fronto-limbic regions as mentioned in the introduction section. To define these regions, the AAL atlas [32] in the WFU PickAtlas toolbox [35] was used. A family-wise error (FWE) correction threshold of p < 0.05 within the SVC was used to determinate significant results from these tests.

#### Region of interest analysis

Because we had an *a priori* hypothesis for the involvement of the amygdala in this task and in the physiopathology of BD, we additionally conducted a region of interest (ROI) analysis to examine the amygdala activity during the task. Left and right amygdala from the AAL atlas were defined through the WFU pickatlas toolbox. For each ROI and each participant, the percent of MR signal change was extracted and the values were included into an analysis of variance (ANOVA) with LR vs. HR trials as within-subject factor and HS vs. EBP as between-subject factor. This analysis allowed assessing differences in amygdala reactivity between groups and according to its possible role in monitoring and resolution of the emotional conflict.

#### Psychophysiological interaction analysis

To assess functional connectivity, we performed a psychophysiological interaction (PPI) analysis. The PPI approach addresses the question of how connectivity with a seed region is modulated by a psychological factor (such as task) [36]. Based on the peak voxels taken from the *Monitoring* contrast in between-group analysis, the most appropriate seed region for the emotional conflict processing appeared to be the right middle frontal cortex (DLPFC; MNI coordinates: [54 23 33], with a 10 mm radius sphere). We used the standard procedure in SPM8: (1) the physiological activity of the seed region was computed as time series of all voxels within the sphere, with the first principal component adjusted for effects of interest (i.e., despiked and denoised); (2) the psychological regressor representing task condition (i.e., LR, HR) was used to determine the condition-specific change in functional connectivity; (3) the PPI variable (i.e., the interaction term) was formed by deconvolving the BOLD signal in order to provide the proper derivation of the interaction term (at neuronal level) [37].

A second GLM analysis that includes the interaction term, the signal extracted from the seed and the experimental factor, was performed. The *t* contrast of [1 0 0] produced statistical image revealing voxels having a significant positive connectivity with the DLPFC during the monitoring of the emotional conflict, whereas the *t* contrast [−1 0 0] revealed regions with negative functional connectivity with the DLPFC during the monitoring. At the second level, the PPI contrast images were entered into one-sample and two-sample *t*-test analyses to evaluate within-group and between-group random effect respectively. The connectivity maps were thresholded at *P* < 0.005 (uncorrected) with a minimum cluster size of 10 voxels. Multiple comparisons were also corrected using SVC to the GLM targeting fronto-limbic regions as mentioned previously.

## Results

### Behavioral results

**Fig 2:**
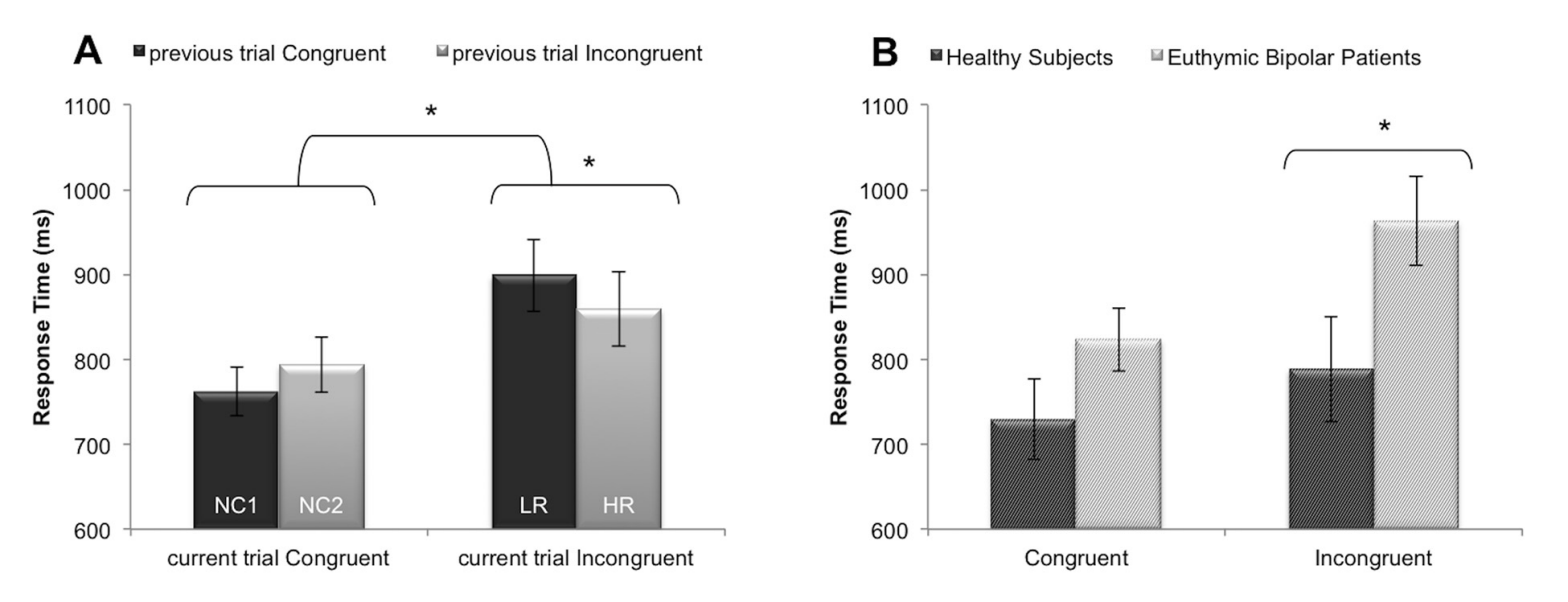
Behavioral performances during word-face emotional Stroop. Panel A: Illustration of behavioral dissociation between conflict monitoring and conflict resolution. The graph shows the mean response time ± SE according to the congruency of the current trial and the congruency of the previous trial. Panel B: Illustration of the increase emotional interference in euthymic bipolar patients. The graph shows the mean response time ± SE according to the group and the congruency of the current trial. *p<0.05. *Abbreviations*: LR: Low conflict resolution; HR: High conflict resolution; NC: No conflict.

All conditions combined, BP patients were marginally slower [*F*(1, 25) = 3.93; *η*_*p*_^*2*^ = 0.14; *P* = 0.06] but in terms of %CR they were comparable to HS [*F*(1, 25) = 0.39; *Η*_*p*_^*2*^ = 0.01; *P* = 0.09]. ANOVAs revealed a significant main effect of emotional conflict conditions in BP and HS for both RT [*F*(3, 75) = 23.73; *Η*_*p*_^*2*^ = 0.49; *P* < 0.001] and %CR [*F*(3, 75) = 9.09; *Η*_*p*_^*2*^ = 0.27; *P* < 0.001]. Planned comparisons confirmed a strong interference effect (incongruent vs. congruent) on both RT [*F*(1, 25) = 39.26; *P* < 0.001] and %CR [*F*(1, 25) = 14.65; *P* < 0.001]. Furthermore, the RT was lower for LR than for HR trials [*F*(1, 25) = 6.84; *P* = 0.01], highlighting the dissociation between monitoring and resolution processes at behavioral level (Fig 2A). A significant Group by Condition interaction was also observed on RT [*F*(3, 75) = 3.87; *Η*_*p*_^*2*^ = 0.13; *P* = 0.01]. Planned comparisons showed that BP were significantly slower than HS for processing incongruent stimuli [*F*(1, 25) = 6.42; *P* = 0.02] (Fig 2B). However, there was no significant difference between BP and HS according to the amount of the conflict, i.e., LR vs HR trials [*F*(1, 25) = 1.61; *P* = 0.22]. Descriptive statistics are summarized in Table 2.

**Table 2:** Behavioral performances for word-face emotional Stroop measured during fMRI.

	LR	HR	NC 1	NC 2
**Response Time (ms)**				
HS	818.69 (238.07)	758.75 (207.83)	716.46 (170.52)	742.37 (170.28)
BP	973.89 (182.83)	953.09 (210.60)	805.60 (120.49)	841.62 (158.00)
**% Correct Responses**				
HS	94.77 (5.26)	93.85 (7.59)	99.36 (1.56)	98.77 (1.92)
BP	93.43 (6.39)	94.57 (6.39)	97.02 (3.81)	98.57 (2.53)

*Note*: Data are reported as Mean (SD). BP: Bipolar patients; HS: Healthy subjects; LR: Low conflict resolution; HR: High conflict resolution; NC1: No conflict 1; NC2: No conflict 2.

### fMRI results

#### Within-group analysis at whole-brain level

**Fig 3:**
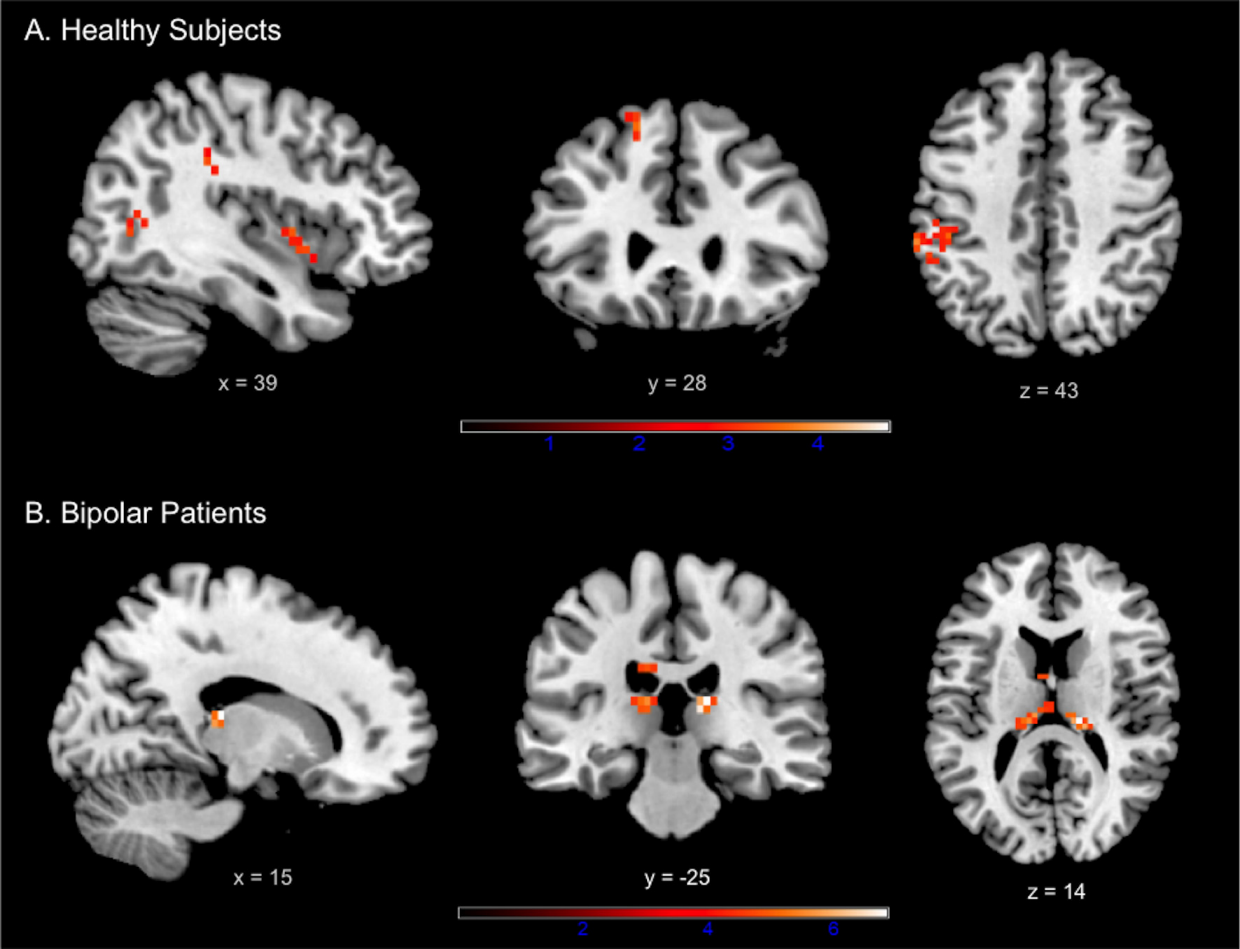
Results of the within-group analyses at whole-brain level during conflict monitoring in (A) healthy subjects and (B) euthymic bipolar patients. Identified regions are projected onto 2D anatomical slices in axial, coronal and sagittal orientations (*p* < 0.001 uncorrected at whole brain level, *p*_FWE_ < 0.05 after small volume correction).

In HS the conflict *Monitoring* contrast (LR > HR) induced activation within the superior frontal, insula, bilateral middle temporal, right inferior temporal, left supramarginal and left middle occipital gyri (Fig 3A, Table 3). In BP, the same contrast elicited brain activation within the bilateral dorsal thalamus only (Fig 3B, Table 3). The opposite contrast (i.e., conflict *Resolution* contrast: HR > LR) did not reveal suprathreshold voxels, neither for HS nor for BP.

**Table 3:** Activation peaks during the emotional conflict monitoring, as revealed by within and between-group analyses.

Lobe	Region	aal-label	H	x	y	z	t	k
**Healthy subjects: Monitoring (LR > HR)**								
Central	Precentral gyrus	PRE	L	-21	25	70	6.39	10
Frontal	Superior frontal gyrus*	F1	L	-15	29	55	5.16	10
Insular	Insula*	IN	R	36	8	-5	3.30	15
Temporal	Middle temporal gyrus	T2	R	54	-19	-5	5.57	18
	Middle temporal gyrus	T2	L	-63	-22	-1	5.00	10
	Inferior temporal gyrus	T3	R	51	-67	-9	5.12	13
Parietal	Inferior parietal/Supramarginal	P2	L	-45	-34	40	6.85	125
Occipital	Middle occipital gyrus	O2	L	-36	-70	10	5.56	21
**Bipolar patients: Monitoring (LR > HR)**								
Sub cortical grey nuclei	Thalamus	THA	R	15	-25	14	4.78	12
	Thalamus	THA	L	-9	-22	14	3.95	24
**Healthy subjects > Bipolar Patients: Monitoring**								
Frontal	Middle frontal gyrus, dorsolateral*	F2	R	54	23	33	3.99	10
	Superior frontal gyrus*	F1	R	6	65	33	5.06	22
Temporal	Middle temporal gyrus	T2	L	-63	-16	-5	4.33	24
Parietal	Inferior parietal/Supramarginal	P2	L	-57	-37	36	4.31	11

*P_FWE_ < 0.05 after small volume correction

LR: Low conflict resolution; HR: High conflict resolution; H: Hemisphere; R: Right; L: Left; k: number of voxels/cluster.

### Between-group analysis at whole-brain level

**Fig 4:**
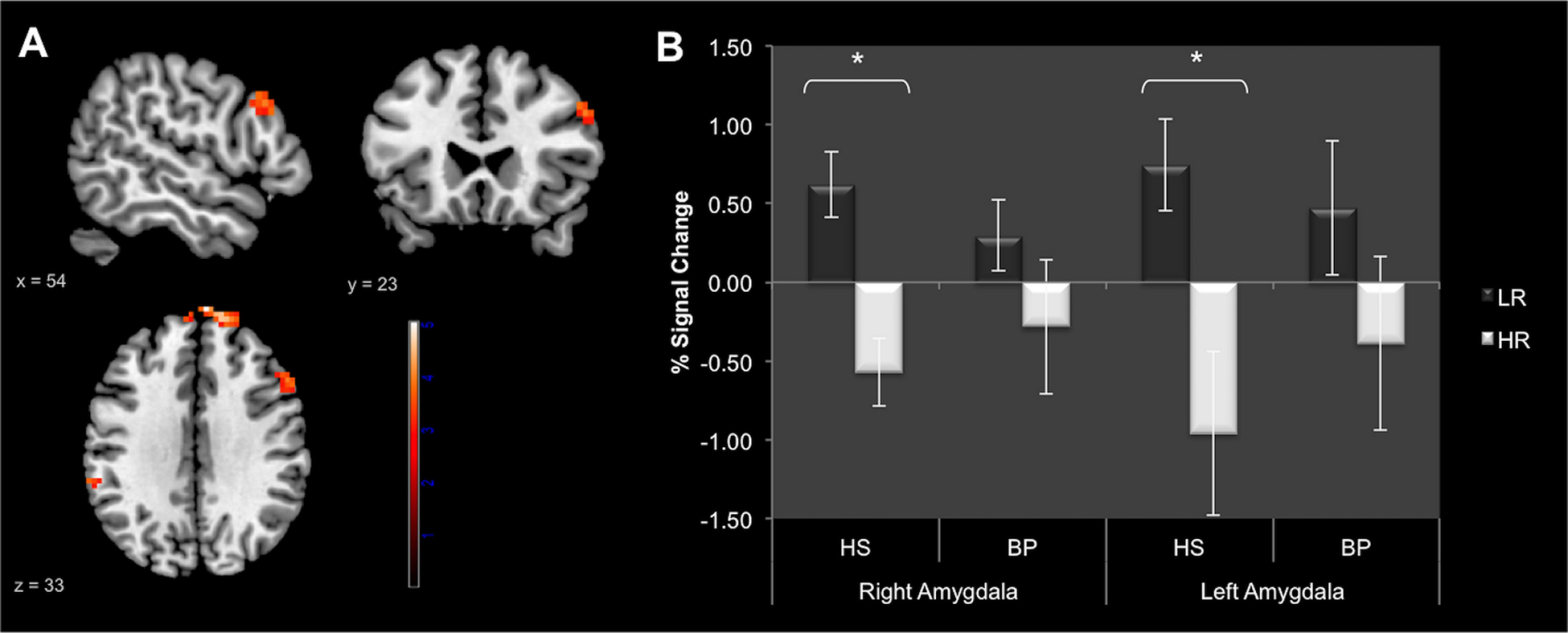
Results provided by between-group analyses. Panel A: Whole-brain comparison in HS vs BP during conflict monitoring (LR > HR) (*p* < 0.001 uncorrected at whole brain level, *p*_FWE_ < 0.05 after small volume correction). Identified regions are projected onto 2D anatomical slices in axial, coronal and sagittal orientations. Panel B: Region of interest analysis focused on bilateral amygdala. The graph shows the mean %MR signal intensity variations ± SE according to the group and the amount of the conflict (LR vs HR). *p<0.05. *Abbreviations*: BP: Bipolar patients; HS: Healthy subjects; LR: Low conflict resolution; HR: High conflict resolution.

The conflict *Monitoring* contrast revealed stronger activation in HS in comparison to BP in frontal regions including the right middle (dorsolateral part) and superior frontal gyri, as well as in the left middle temporal and left supramarginal gyri (Fig 4A, Table 3). No regions were significantly more activated in BP compared to HS. The conflict *Resolution* contrast did not reveal significant differences between groups.

### Region of interest analysis: Amygdala

ROI analysis showed increased activity of bilateral amygdala during the monitoring of emotional conflict. A main effect of *Condition* reflecting higher activity for LR compared to HR trials was observed for both right [*F*(1, 25) = 12.22; *Η*_*p*_^*2*^ = 0.33; *P* = 0.002] and left amygdala [*F*(1, 25) = 7.19; *Η*_*p*_^*2*^ = 0.22; *P* = 0.01]. There was no main Group effect neither for the right [*F*(1, 25) = 0.003; *Η*_*p*_^*2*^ = 0.0001; *P* = 0.95] nor for the left [*F*(1, 25) = 0.11; *Η*_*p*_^*2*^ = 0.004; *P* = 0.74] amygdala. The interaction Group-by-Condition was not significant (right amygdala: [*F*(1, 25) = 1.46; *Η*_*p*_^*2*^ = 0.05; *P* = 0.24]; left amygdala: [*F*(1, 25) = 0.77; *Η*_*p*_^*2*^ = 0.03; *P* = 0.39]). However, as we had specific hypotheses, the monitoring effect on each group was tested. Planned comparison revealed that the activation of bilateral amygdala was significantly higher for LR than for HR trials (i.e., increased during conflict monitoring and decreased during conflict resolution) in HS (right amygdala: [*F*(1, 25) = 10.68; *P* = 0.003]; left amygdala: [*F*(1, 25) = 18.92; *P* = 0.02]), but the difference was not significant in BP (right amygdala: [*F*(1, 25) = 2.71; *P* = 0.11]; left amygdala: [*F*(1, 25) = 1.69; *P* = 0.21]) (Fig 4B).

### PPI results

#### Within-group functional connectivity analysis

**Fig 5:**
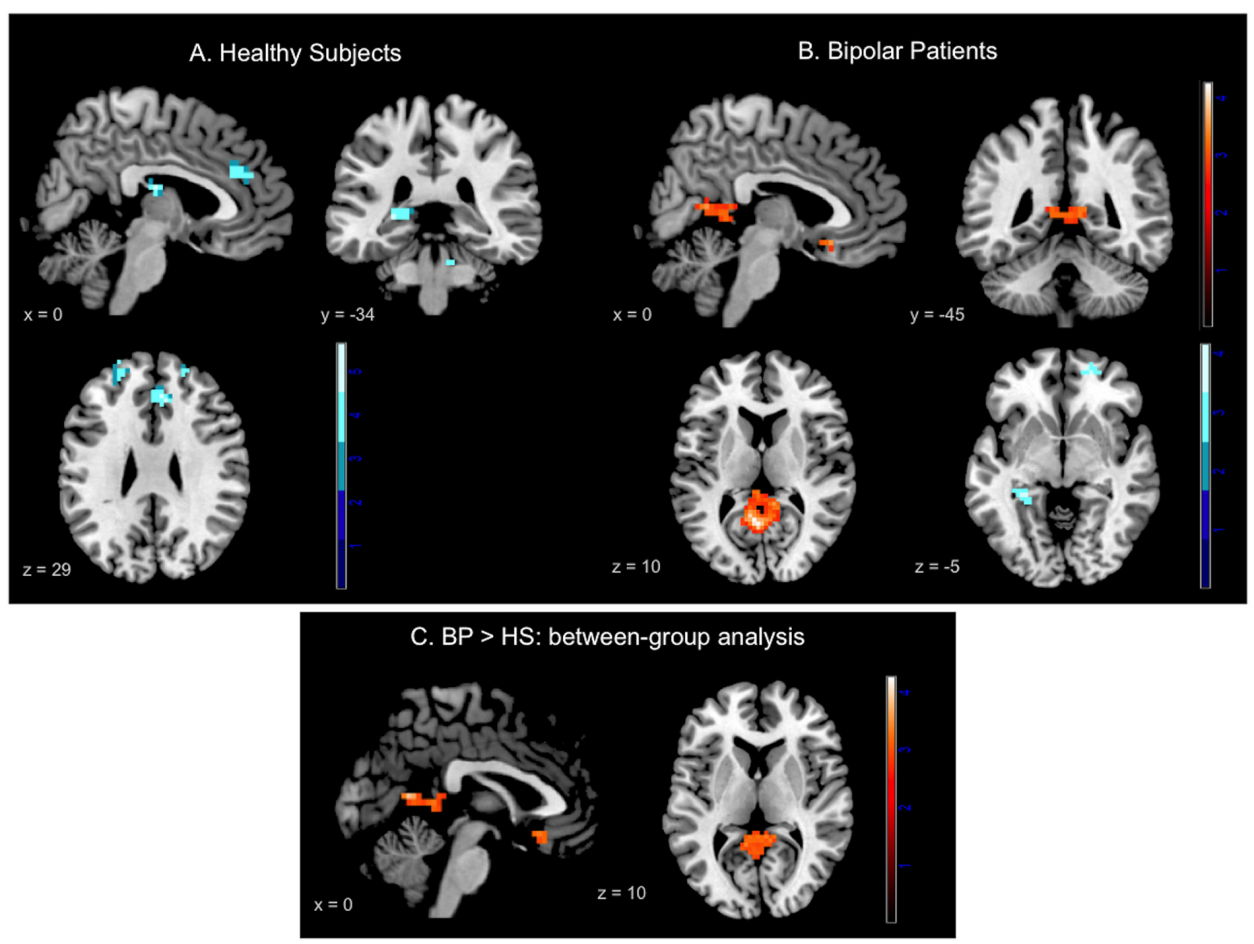
Psychophysiological interaction results. Panel A: Results provided by “within group” analysis in healthy subjects; Panel B: Results provided by “within group” analysis in bipolar patients; Panel C: Results provided by the “between-group” analysis in Bipolar patients vs. Healthy subjects. Red-scale areas represent regions showing positive connectivity with the right dorsolateral prefrontal cortex; Blue-scale areas represent regions showing negative connectivity with the right dorsolateral prefrontal cortex. Identified regions are projected onto 2D anatomical slices in axial, coronal and sagittal orientations (*p* < 0.005 uncorrected at whole brain level, *p*_FWE_ < 0.05 after small volume correction).

As the conflict *Resolution* contrast did not elicited significant results, the PPI analyses were only conducted on the conflict *Monitoring* contrast. Within-group analysis for HS revealed significant negative connectivity between the right DLPFC and bilateral superior and middle frontal gyri as well as with the right middle temporal gyrus. More interestingly, HS also showed negative connectivity between the right DLPFC and some areas of the DMN, such as the ACC and the left hippocampus (Fig 5A, Table 4). No region showed significant positive connectivity with the right DLPFC in HS. In BP, the analysis revealed significant negative connectivity between the right DLPFC and the left hippocampus solely. Moreover, BP showed positive connectivity between the right DLPFC and three areas of the DMN, i.e., the subgenual ACC (sgACC), the right angular gyrus and a cluster that encompassed the precuneus and the PCC bilaterally (Fig 5B, Table 4).

**Table 4:** Psychophysiological interaction results of within and between-group analyses.

Lobe	Region	aal-label	H	x	y	z	t	k
**Healthy subjects: negative regression**								
Frontal	Superior frontal gyrus*	F1	R	18	29	59	4.35	19
	Superior frontal gyrus*	F1	L	-12	32	59	5.58	52
	Middle frontal gyrus*	F2	R	24	50	25	4.07	15
	Middle frontal gyrus*	F2	L	-21	50	29	4.24	21
Limbic	Anterior cingulate gyrus*	AC	R/L	9	32	29	5.52	44
	Hippocampus*	HIP	L	-27	-34	3	5.26	23
Temporal	Middle temporal gyrus	T2	R	57	-43	6	5.63	11
**Bipolar patients: negative regression**								
Limbic	Hippocampus*	HIP	L	-30	-37	-5	3.93	14
**Bipolar patients: positive regression**								
Limbic	Anterior cingulate/gyrus rectus*	AC/GR	R/L	0	20	-13	4.04	23
Parietal	Precuneus*	PQ	L	-9	-52	10	3.99	49
	Angular gyrus	AG	R	45	-37	33	3.74	33
**Bipolar patients > Healthy Subjects: positive regression**								
Frontal	Superior frontal gyrus*	F1	L	-21	65	21	3.95	17
Limbic	Anterior cingulate/gyrus rectus*	AC/GR	R/L	3	23	-13	3.54	11
Parietal	Precuneus/Posterior cingulate gyrus*	PQ/PC	L/L	-6	-55	10	4.27	109

*P_FWE_ < 0.05 after small volume correction

LR: Low conflict resolution; HR: High conflict resolution; H: Hemisphere; R: Right; L: Left; k: number of voxels/cluster

#### Between-group functional connectivity analysis

The between-group comparison revealed greater positive connectivity in BP compared to HS between the right DLPFC and three major hubs of the DMN, i.e., the sgACC, the precuneus/PCC and the superior frontal gyrus (Fig 5C, Table 4). There were no regions eliciting significantly greater connectivity in HS compared to BP.

## Discussion

The present study examined behavioral and cerebral correlates of emotional conflict processing in euthymic BP. We used a word-face emotional Stroop designed to disentangle between cerebral mechanisms involved in the monitoring and in the resolution of the emotional conflict. The use of this task allowed implicit assessment of automatic emotion regulation processing in euthymic BP. At behavioral level, (i) we replicated prior findings showing increased reaction time for low conflict resolution trials compared to high resolution trials; (ii) we showed increased emotional inference effect in BP in comparison to HS. At cerebral level, we demonstrated that (i) compared to HS, BP presented decreased activation of the right DLPFC during conflict monitoring; (ii) bilateral amygdala were significantly activated for LR trials and deactivated for HR trials in HS but not in BP; (iii) the right DLPFC was significantly negatively connected to areas of the DMN in HS but positively connected to major hubs of this network in BP during monitoring of emotional conflict. This difference appeared also in the between-group comparison, which revealed that the right DLPFC was significantly more connected to the sgACC and the PCC in BP compared to HS.

Commenting on behavioral results, we reproduced previous findings that demonstrated dissociation between low conflict resolution (i.e., LR) and high conflict resolution trials. Indeed, processing of incongruent stimuli preceded by congruent stimuli leads to higher interference and slower responses (increased RT), and processing of incongruent stimuli preceded by incongruent stimuli facilitates responses (faster RT) [16, 19, 20]. Moreover, we showed that, in addition to a general slowdown, BP were also significantly slower than HS in processing incongruent stimuli, suggesting increase emotional interference in BP. This is in agreement with neuropsychological studies which highlighted increased Stroop effect in BP in the original color-word Stroop task, even during euthymic state [38, 39]. This suggests that BP show persistent deficits in selective attention and inhibitory control. Using the emotional [17, 18] or the color-word Stroop task [40, 41], previous fMRI studies did not reveal increased behavioral interference effect in BP. This suggests that the control of the proportion of HR and LR trials may be crucial to precisely evaluate conflict processing in BP. Nonetheless, we did not obtain significant differences in terms of RT between BP and HS for processing LR vs. HR stimuli. This may be due to the small power of our study for behavioral comparisons.

However, at the cerebral level, in comparison to HS, BP presented lower DLPFC activation during conflict monitoring. The DLPFC activity is supposed to reflect task difficulty, selective attention and high-memory load recruitment for processing incongruent stimuli during interference task and for task switching [42, 43]. Consequently, diminished DLPFC activation in BP during conflict monitoring could underlie the difficulty for these patients to manage the interference arising from two sources of information (valence of word vs. valence of facial expression). This could have led to increased interference effect at the behavioral level. Otherwise, the role of the DLPFC in emotion regulation processes was also underlined, in particular for voluntary emotion regulation and cognitive reappraisal sub-processes [10, 44]. In addition, increased DLPFC activity was significantly associated with decreased amygdala activation in voluntary emotion regulation task [45].

In our task, the ROI analysis focused on the amygdala revealed significantly higher bilateral activity for LR versus HR trials (i.e., during the monitoring process) in HS. This result is in accordance with Etkin et al. [16] who also pointed out the increased right amygdala activity during conflict monitoring. Moreover, Etkin et al. [16], demonstrated that the activity of amygdala was attenuated during the resolution by a top-down control exerted by the rostral ACC. Interestingly, in our study it appears that, in BP, the amygdala activity did not differ between LR and HR trials. Indeed, BP showed a lack of amygdala activation for LR trials (i.e., during the monitoring) but also less deactivation for HR trials (i.e., during the resolution). We can assume a deficit of the top-down control exerted by the DLPFC during the monitoring leads to less inhibition/deactivation of the amygdala during the conflict resolution.

Therefore, we investigated the functional connectivity of the right DLPFC during the task by using the PPI analysis. Surprisingly, in HS, the DLPFC was negatively connected to main regions of the DMN, i.e., the bilateral superior and middle frontal gyri, the ACC and the left hippocampus [46, 47]. In the context of the resting-state paradigm, the activation of the MPFC/ACC is supposed to reflect internally- generated thoughts and self-referential processing [48]; as regard to hippocampus activation, it is intended to underlie episodic memory processes [49]. In the current study, the negative connectivity of the DLPFC with the ACC and the hippocampus obtained in HS would reflect the disengagement of the DMN during the conflict monitoring. Indeed to solve the conflict, participants need to remain attentive and stay focused on the stimuli to do not be distracted by incongruent words, which requires to strongly inhibit the mind-wandering processes. Unexpectedly, in BP, the right DLPFC was positively connected to other DMN regions, such as the PCC/Precuneus and the sub-genual ACC. In the between-group comparison, these connections were also significantly stronger in BP compared to HS. One possible explanation could be that BP experience difficulties to disengage the DMN at the time of conflict monitoring. This assumption is supported by results of our previous resting-state study suggesting abnormal decoupling (i.e., a lack of negative connectivity) between the ventral MPFC and the right DLPFC in euthymic BP [23]. We proposed that, even at rest, BP had difficulties to switch between internal-mode of information processing (reflected by the activation of the DMN) and external-mode of information processing (reflected by the activity of the DLPFC, which could be included in a larger “task-positive network”—TPN) [50, 51]. Taken together, the results provided by our previous resting-state study and the results obtained in the current study suggest abnormal coupling between DMN and TPN in BP, both at rest and during a task requiring strong cognitive effort, such as emotion conflict processing. We assume that poorer performances of the BP group during the task (i.e., increased emotional interference) would be due to a lack of disengagement of the attention on self, which would have led in turn to less effective conflict management. This explanation is supported by the positive connectivity between the DLPFC and the subgenual part of the ACC observed in BP. Indeed, this brain area is known to be involved in mental ruminations and depression symptoms in patients with unipolar depression (UD) [52–54]. Furthermore, abnormally increased connectivity between the DMN and the sgACC was reported in patients with UD and supposed to be related to ruminations during the resting-state [55]. Consequently, residual depressive symptoms in euthymic BP may result of this abnormally increased connectivity between the DLPFC and the sgACC during the emotional conflict processing. Future studies will assess whether these abnormalities of functional connectivity are related to mind-wandering particularities in euthymic BP.

At least three limitations of this study should be mentioned. First, statistical thresholds used for whole-brain analyses were not corrected for multiple comparisons at a whole-brain level. The lack of power to detect significant corrected results could be due to the small sample size of study. Consequently, generalization of our findings is limited. Second, the majority of patients included in the study were medicated and different combinations of treatment might have influenced the results but they were too heterogeneous to assess their effects. Third, the clinical subtypes of patients included in this study were various, both BD I and BD II, which could limit the specificity of our findings. Future studies focused on each clinical subtype of BD are needed to elucidate the precise pathophysiological mechanisms of BD.

However, this is the first study that aimed to disentangle different sub-processes of emotional conflict processing in BD. We provide behavioral and cerebral evidence for impairment of emotion conflict monitoring and resolution in euthymic BP. Our main findings highlight dysfunction of the right DLPFC in BP during conflict monitoring (generation), which could be responsible for poorer performances in patients in response to emotional interference. Moreover, BP showed lack of deactivation of bilateral amygdala during the conflict resolution, potentially related to insufficient top-down control of prefrontal region overwhelmed by the amount of the conflict. In addition, the right DLPFC was abnormally positively connected to the sgACC and the PCC/Precuneus during the conflict monitoring, thus reflecting the inability of BP to withdraw their attention from themselves and redirect it toward the task/environment. Further studies are required to better understand the dynamic of connectivity changes between the lateral prefrontal, medial prefrontal and limbic regions in BP during voluntary and automatic emotion regulation processing.

Clinical trial registration number: NCT01821469

## References

[1] Henry C, Van den Bulke D, Bellivier F, Roy I, Swendsen J, M'Baïlara K, et al Affective lability and affect intensity as core dimensions of bipolar disorders during euthymic period. Psychiatry Res. 2008;159(1):1–6. 18295902 10.1016/j.psychres.2005.11.016

[2] M'Bailara K, Demotes-Mainard J, Swendsen J, Mathieu F, Leboyer M, Henry C. Emotional hyper-reactivity in normothymic bipolar patients. Bipolar Disord. 2009;11(1):63–9. 10.1111/j.1399-5618.2008.00656.x 19133967

[3] Martínez-Arán A, Vieta E, Colom F, Torrent C, Sánchez-Moreno J, Reinares M, et al Cognitive impairment in euthymic bipolar patients: implications for clinical and functional outcome. Bipolar Disord. 2004;6(3):224–32. 15117401 10.1111/j.1399-5618.2004.00111.x

[4] Manove E, Levy B. Cognitive impairment in bipolar disorder: an overview. Postgrad Med. 2010;122(4):7–16. 10.3810/pgm.2010.07.2170 20675966

[5] Strakowski SM, Adler CM, Almeida J, Altshuler LL, Blumberg HP, Chang KD, et al The functional neuroanatomy of bipolar disorder: a consensus model. Bipolar Disord. 2012;14(4):313–25. 10.1111/j.1399-5618.2012.01022.x 22631617 PMC3874804

[6] Phillips ML, Swartz HA. A Critical Appraisal of Neuroimaging Studies of Bipolar Disorder: Toward a New Conceptualization of Underlying Neural Circuitry and a Road Map for Future Research. Am J Psychiatry. 2014;in press. 10.1176/appi.ajp.2014.13081008 PMC4119497 24626773

[7] Chen CH, Suckling J, Lennox BR, Ooi C, Bullmore ET. A quantitative meta-analysis of fMRI studies in bipolar disorder. Bipolar Disord. 2011;13(1):1–15. 10.1111/j.1399-5618.2011.00893.x 21320248

[8] Houenou J, Frommberger J, Carde S, Glasbrenner M, Diener C, Leboyer M, et al Neuroimaging-based markers of bipolar disorder: Evidence from two meta-analyses. J Affect Disord. 2011. 10.1016/j.jad.2011.03.016 21470688

[9] Townsend JD, Altshuler LL. Emotion processing and regulation in bipolar disorder: a review. Bipolar Disord. 2012;14(4):326–39. 10.1111/j.1399-5618.2012.01021.x 22631618

[10] Phillips ML, Ladouceur CD, Drevets WC. A neural model of voluntary and automatic emotion regulation: implications for understanding the pathophysiology and neurodevelopment of bipolar disorder. Mol Psychiatry. 2008;13(9):833–57. 10.1038/mp.2008.65 PMC2745893 18574483

[11] Whalen PJ, Bush G, McNally RJ, Wilhelm S, McInerney SC, Jenike MA, et al The emotional counting Stroop paradigm: a functional magnetic resonance imaging probe of the anterior cingulate affective division. Biol Psychiatry. 1998;44(12):1219–28. 9861465 10.1016/s0006-3223(98)00251-0

[12] Blair KS, Smith BW, Mitchell DGV, Morton J, Vythilingam M, Pessoa L, et al Modulation of emotion by cognition and cognition by emotion. Neuroimage. 2007;35(1):430–40. 17239620 10.1016/j.neuroimage.2006.11.048 PMC1862681

[13] Lagopoulos J, Malhi GS. A functional magnetic resonance imaging study of emotional Stroop in euthymic bipolar disorder. Neuroreport. 2007;18(15):1583 17885606 10.1097/WNR.0b013e3282efa07a

[14] Malhi GS, Lagopoulos J, Sachdev PS, Ivanovski B, Shnier R. An emotional Stroop functional MRI study of euthymic bipolar disorder. Bipolar Disord. 2005;7:58–69. 16225562 10.1111/j.1399-5618.2005.00255.x

[15] Stroop JR. Studies of interference in serial verbal reactions. J Exp Psychol. 1935;18:643–62.

[16] Etkin A, Egner T, Peraza DM, Kandel ER, Hirsch J. Resolving emotional conflict: a role for the rostral anterior cingulate cortex in modulating activity in the amygdala. Neuron. 2006;51(6):871–82. 16982430 10.1016/j.neuron.2006.07.029

[17] Favre P, Baciu M, Pichat C, De Pourtalès M-A, Fredembach B, Garçon S, et al Modulation of fronto-limbic activity by the psychoeducation in euthymic bipolar patients. A functional MRI study. Psychiatry Res Neuroimaging. 2013;214(3):285–95. 10.1016/j.pscychresns.2013.07.007 24156926

[18] Rey G, Desseilles M, Favre S, Dayer A, Piguet C, Aubry J-M, et al Modulation of brain response to emotional conflict as a function of current mood in bipolar disorder: Preliminary findings from a follow-up state-based fMRI study. Psychiatry Res Neuroimaging. 2014;in press. 10.1016/j.pscychresns.2014.04.016 24862389

[19] Kerns JG, Cohen JD, MacDonald AW, Cho RY, Stenger VA, Carter CS. Anterior cingulate conflict monitoring and adjustments in control. Science. 2004;303(5660):1023–6. 14963333 10.1126/science.1089910

[20] Egner T, Hirsch J. The neural correlates and functional integration of cognitive control in a Stroop task. Neuroimage. 2005;24(2):539–47. 15627596 10.1016/j.neuroimage.2004.09.007

[21] Braver TS. The variable nature of cognitive control: a dual mechanisms framework. Trends in cognitive sciences. 2012;16(2):106–13. 10.1016/j.tics.2011.12.010 22245618 PMC3289517

[22] Botvinick MM, Braver TS, Barch DM, Carter CS, Cohen JD. Conflict monitoring and cognitive control. Psychol Rev. 2001;108(3):624 11488380 10.1037/0033-295x.108.3.624

[23] Favre P, Baciu M, Pichat C, Bougerol T, Polosan M. fMRI evidence for abnormal resting-state functional connectivity in euthymic bipolar patients. J Affect Disord. 2014;165:182–9. 10.1016/j.jad.2014.04.054 24882198

[24] Montgomery SA, Asberg M. A new depression scale designed to be sensitive to change. Br J Psychiatry. 1979;134(4):382–9. 444788 10.1192/bjp.134.4.382

[25] Pellet J, Bobon D, Mormont I, Lang F, Massardier A. Etude princeps de validation française de la MADRS, sous échelle de dépression de la CPRS Compte rendu du Congrès de Psychiatrie et de Neurologie de langue Française. Paris, Masson; 1981.

[26] Young RC, Biggs JT, Ziegler VE, Meyer DA. A rating scale for mania: reliability, validity and sensitivity. Br J Psychiatry. 1978;133(5):429–35. 728692 10.1192/bjp.133.5.429

[27] Favre S, Aubry JM, Gex-Fabry M, Ragama-Pardos E, McQuillan A, Bertschy G. Translation and validation of a French version of the Young Mania Rating Scale (YMRS). L'Encephale. 2003;29(6):499 15029084

[28] First MB, Spitzer RL, Gibbon M, Williams JBW. Structured Clinical Interview for DSM-IV-TR Axis I Disorders: SCID-I, Patients Edition (SCID-I/P). New York: Biometrics Research Department, New York State Psychiatric Institute; 2002.

[29] Ekman P, Friesen WV. Pictures of facial affect. Palo Alto: Consulting Psychologists Press; 1976.

[30] Beaupré MG, Hess U. Cross-cultural emotion recognition among Canadian ethnic groups. Journal of Cross-Cultural Psychology. 2005;36(3):355–70.

[31] Friston KJ, Zarahn E, Josephs O, Henson RNA, Dale AM. Stochastic designs in event-related fMRI. Neuroimage. 1999;10(5):607–19. 10547338 10.1006/nimg.1999.0498

[32] Tzourio-Mazoyer N, Landeau B, Papathanassiou D, Crivello F, Etard O, Delcroix N, et al Automated anatomical labeling of activations in SPM using a macroscopic anatomical parcellation of the MNI MRI single-subject brain. Neuroimage. 2002;15(1):273–89. 11771995 10.1006/nimg.2001.0978

[33] Friston KJ, Holmes AP, Worsley KJ, Poline JP, Frith CD, Frackowiak RSJ. Statistical parametric maps in functional imaging: a general linear approach. Hum Brain Mapp. 1995;2(4):189–210.

[34] Friston KJ, Fletcher P, Josephs O, Holmes A, Rugg MD, Turner R. Event-related fMRI: characterizing differential responses. Neuroimage. 1998;7(1):30–40. 9500830 10.1006/nimg.1997.0306

[35] Maldjian JA, Laurienti PJ, Kraft RA, Burdette JH. An automated method for neuroanatomic and cytoarchitectonic atlas-based interrogation of fMRI data sets. Neuroimage. 2003;19(3):1233 12880848 10.1016/s1053-8119(03)00169-1

[36] Friston KJ, Buechel C, Fink GR, Morris J, Rolls E, Dolan RJ. Psychophysiological and modulatory interactions in neuroimaging. Neuroimage. 1997;6(3):218–29. 9344826 10.1006/nimg.1997.0291

[37] Gitelman DR, Penny WD, Ashburner J, Friston KJ. Modeling regional and psychophysiologic interactions in fMRI: the importance of hemodynamic deconvolution. Neuroimage. 2003;19(1):200–7. 12781739 10.1016/s1053-8119(03)00058-2

[38] Robinson LJ, Thompson JM, Gallagher P, Goswami U, Young AH, Ferrier IN, et al A meta-analysis of cognitive deficits in euthymic patients with bipolar disorder. J Affect Disord. 2006;93(1):105–15. 16677713 10.1016/j.jad.2006.02.016

[39] Bora E, Yucel M, Pantelis C. Cognitive endophenotypes of bipolar disorder: a meta-analysis of neuropsychological deficits in euthymic patients and their first-degree relatives. J Affect Disord. 2009;113(1):1–20. 18684514 10.1016/j.jad.2008.06.009

[40] Pompei F, Jogia J, Tatarelli R, Girardi P, Rubia K, Kumari V, et al Familial and disease specific abnormalities in the neural correlates of the Stroop Task in Bipolar Disorder. Neuroimage. 2011;56(3):1677–84. 10.1016/j.neuroimage.2011.02.052 21352930

[41] Kronhaus DM, Lawrence NS, Williams AM, Frangou S, Brammer MJ, Williams SCR, et al Stroop performance in bipolar disorder: further evidence for abnormalities in the ventral prefrontal cortex. Bipolar Disord. 2006;8(1):28–39. 16411978 10.1111/j.1399-5618.2006.00282.x

[42] Duncan J, Owen AM. Common regions of the human frontal lobe recruited by diverse cognitive demands. Trends Neurosci. 2000;23(10):475–83. 11006464 10.1016/s0166-2236(00)01633-7

[43] Fuster JM. The prefrontal cortex- an Update:Time is of the essence. Neuron. 2001;30(2):319–33. 11394996 10.1016/s0896-6273(01)00285-9

[44] Ochsner KN, Gross JJ. The cognitive control of emotion. Trends in cognitive sciences. 2005;9(5):242–9. 15866151 10.1016/j.tics.2005.03.010

[45] Walter H, von Kalckreuth A, Schardt D, Stephan A, Goschke T, Erk S. The temporal dynamics of voluntary emotion regulation. PLoS ONE. 2009;4(8):e6726–e. 10.1371/journal.pone.0006726 21949675 PMC3175755

[46] Raichle ME, MacLeod AM, Snyder AZ, Powers WJ, Gusnard DA, Shulman GL. A default mode of brain function. Proc Natl Acad Sci U S A. 2001;98(2):676–82. 11209064 10.1073/pnas.98.2.676 PMC14647

[47] Raichle ME, Snyder AZ. A default mode of brain function: a brief history of an evolving idea. Neuroimage. 2007;37(4):1083–90. 17719799 10.1016/j.neuroimage.2007.02.041

[48] Gusnard DA, Akbudak E, Shulman GL, Raichle ME. Medial prefrontal cortex and self-referential mental activity: relation to a default mode of brain function. Proceedings of the National Academy of Sciences. 2001;98(7):4259–64. 10.1073/pnas.071043098 PMC31213 11259662

[49] Buckner RL, Andrews-Hanna JR, Schacter DL. The brain's default network: anatomy, function, and relevance to disease Ann N Y Acad Sci. 2008;1124(1):1–38. 18400922 10.1196/annals.1440.011

[50] Corbetta M, Shulman GL. Control of goal-directed and stimulus-driven attention in the brain. Nat Rev Neurosci. 2002;3(3):215–29. 10.1038/nrn755 11994752

[51] Fox MD, Snyder AZ, Vincent JL, Corbetta M, Van Essen DC, Raichle ME. The human brain is intrinsically organized into dynamic, anticorrelated functional networks. Proc Natl Acad Sci U S A. 2005;102(27):9673–8. 15976020 10.1073/pnas.0504136102 PMC1157105

[52] Lemogne C, le Bastard G, Mayberg H, Volle E, Bergouignan L, Lehéricy S, et al In search of the depressive self: extended medial prefrontal network during self-referential processing in major depression. Soc Cogn Affect Neurosci. 2009;4(3):305–12. 10.1093/scan/nsp008 19307251 PMC2728628

[53] Nejad AB, Fossati P, Lemogne C. Self-referential processing, rumination, and cortical midline structures in major depression. Front Hum Neurosci. 2013;7:666 10.3389/fnhum.2013.00666 24124416 PMC3794427

[54] Mayberg HS, Liotti M, Brannan SK, McGinnis S, Mahurin RK, Jerabek PA, et al Reciprocal limbic-cortical function and negative mood: converging PET findings in depression and normal sadness. Am J Psychiatry. 1999;156(5):675–82. 10327898 10.1176/ajp.156.5.675

[55] Greicius MD, Flores BH, Menon V, Glover GH, Solvason HB, Kenna H, et al Resting-state functional connectivity in major depression: abnormally increased contributions from subgenual cingulate cortex and thalamus. Biol Psychiatry. 2007;62(5):429–37. 17210143 10.1016/j.biopsych.2006.09.020 PMC2001244

